# Triggering Receptors Expressed on Myeloid Cells 1 : Our New Partner in Human Oncology?

**DOI:** 10.3389/fonc.2022.927440

**Published:** 2022-07-08

**Authors:** Marie Muller, Vincent Haghnejad, Anthony Lopez, Angelica Tiotiu, Stéphane Renaud, Marc Derive, Jean-Pierre Bronowicki

**Affiliations:** ^1^ Department of Gastroenterology, Nancy University Hospital, University of Lorraine, Nancy, France; ^2^ Department of Pulmonology, Nancy University Hospital, University of Lorraine, Nancy, France; ^3^ Department of Thoracic Surgery, Nancy University Hospital, University of Lorraine, Nancy, France; ^4^ INOTREM, University of Lorraine, Nancy, France; ^5^ Inserm U1256 « Nutrition – Genetics and exposure to environmental risks - NGERE », Nancy, France

**Keywords:** inflammation, cancer, hepatocellular carcinoma, lung cancer, TREM-1

## Abstract

Inflammation is recognized as one of the hallmarks of cancer. Indeed, strong evidence indicates that chronic inflammation plays a major role in oncogenesis, promoting genome instability, epigenetic alterations, proliferation and dissemination of cancer cells. Mononuclear phagocytes (MPs) have been identified as key contributors of the inflammatory infiltrate in several solid human neoplasia, promoting angiogenesis and cancer progression. One of the most described amplifiers of MPs pro-inflammatory innate immune response is the triggering receptors expressed on myeloid cells 1 (TREM-1). Growing evidence suggests TREM-1 involvement in oncogenesis through cancer related inflammation and the surrounding tumor microenvironment. In human oncology, high levels of TREM-1 and/or its soluble form have been associated with poorer survival data in several solid malignancies, especially in hepatocellular carcinoma and lung cancer. TREM-1 should be considered as a potential biomarker in human oncology and could be used as a new therapeutic target of interest in human oncology (TREM-1 inhibitors, TREM-1 agonists). More clinical studies are urgently needed to confirm TREM-1 (and TREM family) roles in the prognosis and the treatment of human solid cancers.

## Introduction

The hallmarks of cancer include several biological abilities that are acquired during the multistep process of human tumors (ie: proliferative signaling, evading growth suppressors, angiogenesis induction, escape and metastasis activation) (1, 2). Among them, inflammation is now recognized as a defining hallmark of cancer (2). Indeed, strong evidence indicate that chronic inflammation plays a major role in oncogenesis, promoting genome instability, epigenetic alterations, proliferation and dissemination of cancer cells (3, 4). Moreover, necrotic cell death from tumors generates proinflammatory signals (ie: IL-1α) in the proximal tissue microenvironment, in opposition to apoptosis and autophagy (2). Necrotic cells are able to enroll inflammatory elements from the immune system, including mononuclear phagocytes (MPs), which contribute to angiogenesis, invasiveness and therefore cancer progression (5). MPs have been identified as key contributors of the inflammatory infiltration in several solid human cancers (6, 7). They can be actively recruited from the circulation to tumor fields by tumor-related agents as primary monocytes, which differentiate into tumor-associated macrophages (TAMs) or tumor-associated dendritic cells (TADCs) (8, 9). The most described enhancer of MPs pro-inflammatory innate immune response are the triggering receptors expressed on myeloid cells (TREM), especially TREM-1. TREM proteins are a community of cell surface receptors mostly expressed on myeloid cells (10). The most described ligands of TREM-1 are pathogen-associated molecular patterns (PAMPs) and damage associated molecular patterns (DAMPs, endogenous ligands released after sterile tissue injury). More recently, heat shock protein 70-kDA (HSP70), peptidoglycan recognition receptor 1 (PGLYRP1) and platelets were also described as potential TREM-1 ligands (11).

Engagement of TREM-1 plays a crucial role in the modulation of inflammation. Indeed, it leads to an increase of tumor necrosis factor α (TNFα) secretion by monocytes, IL-8 and monocyte chemoattractant protein 1. The release of these elements induces neutrophil degranulation and the secretion of myeloperoxidase and nitric oxide (12, 13). Beside its membrane-anchored form, TREM-1 is released as a soluble protein (sTREM-1) upon its activation by proteolytic cleavage, and sTREM-1 is a useful biomarker of the activation of the TREM-1 pathway (14).

The TREM family has been mainly investigated in severe inflammation (15). However, it is now well known that TREM-1 is highly expressed in some tumoral tissues, as hepatocellular carcinoma and lung carcinoma. Growing evidence suggests TREM-1 involvement in oncogenesis through cancer-associated inflammation and the tumor microenvironment (TME) (13).

In this review, we describe current knowledge about TREM-1 in human solid tumors.

**Figure 1:**
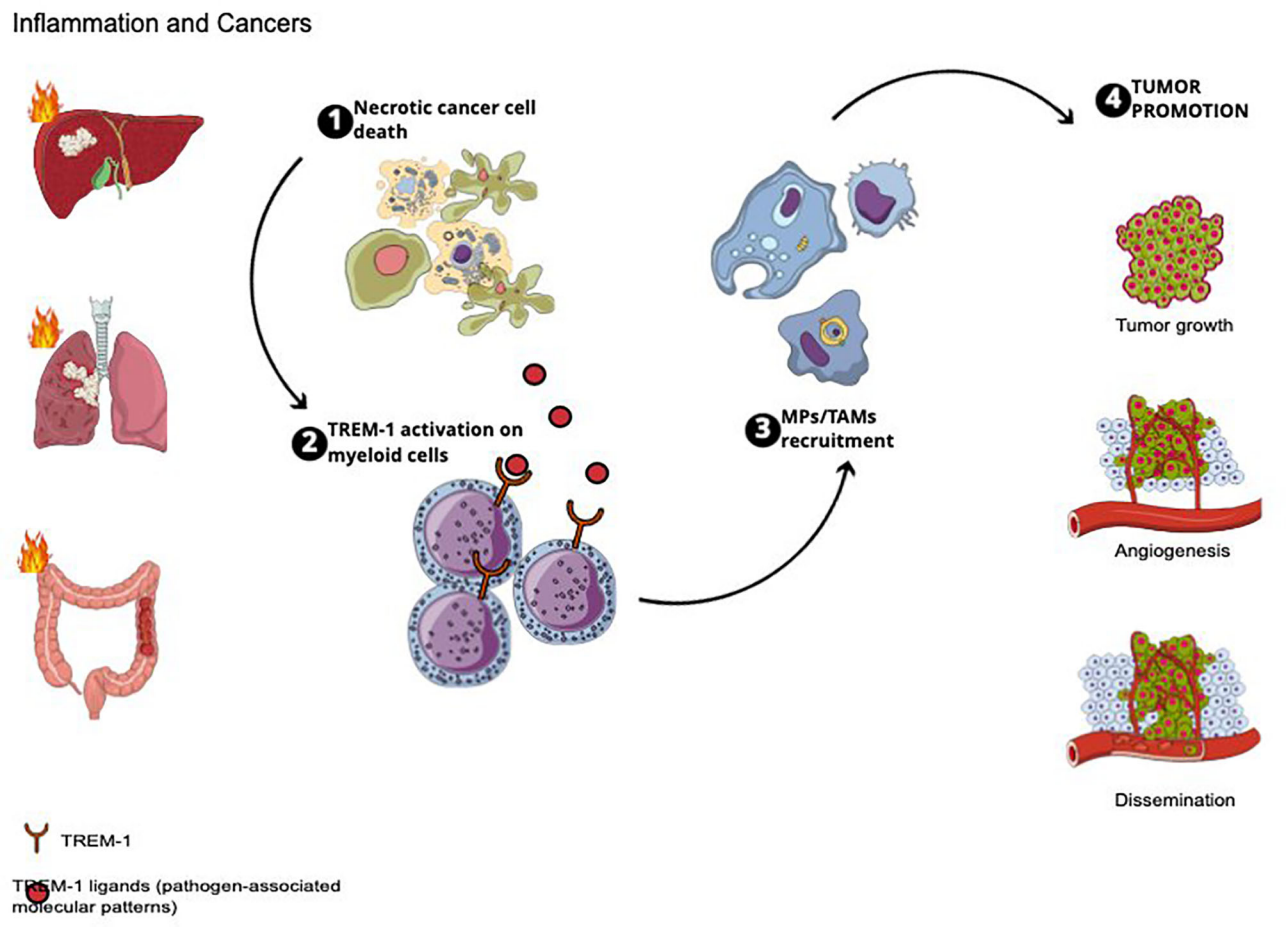
TREM-1 implications in cancer promotion.

TREM-1 implications in cancer promotion are summarized in **Figure 1**.

**Figure 2:**
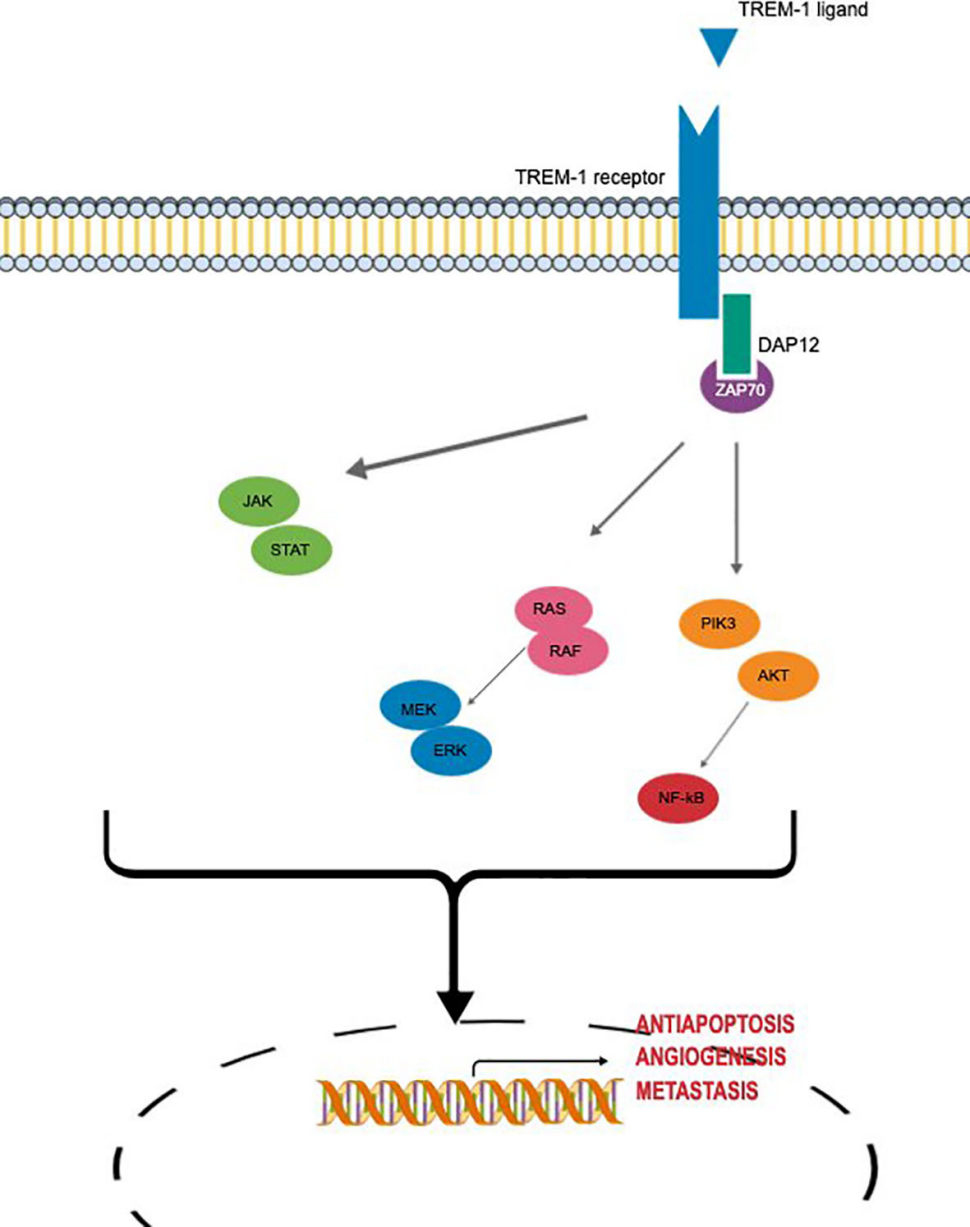
Main molecular signaling of TREM-1 involved in oncogenesis.

Main molecular signaling of TREM-1 involved in oncogenesis are summarized in **Figure 2**.

## TREM-1 in Digestive Oncology

### Hepatocellular Carcinoma

Human hepatocellular carcinoma (HCC) is a very strong example of inflammation-associated cancer. HCC quietly occurs in the setting of chronic inflammation due to an exposition to infectious pathogens or toxic compounds (16). Indeed, in most cases (> 90%), human HCC occurs in the setting of chronic inflammation or cirrhosis. In both conditions, hepatocytes are destroyed and resident inflammatory cells [Kupffer cells (KC)], as well as other inflammatory cells (monocytes, neutrophils), are triggered to release cytokines that lead to a counterbalanced expansion of the surviving hepatocytes (17).

The role of TREM-1 has been recently studied in 3 distinct works. Ten years ago, Liao et al. (18) first investigated TREM-1 expression in human HCC. They used immunohistochemistry (IHC) to assess peritumoral and intratumoral TREM-1 expression on tissue microarray from 240 patients with HCC. They demonstrated that peritumoral TREM-1 expression was significantly associated with vascular invasion (p < 0.001), tumor size (p = 0.001) and high TNM stage (p < 0.001) (18). In addition, high density of peritumoral TREM-1 was associated with elevated risk of recurrence (p = 0.008) and poor overall survival (OS) (p < 0.001) (18). They also determined soluble TREM-1 (sTREM-1) levels (using ELISA) from the plasma of 92 patients operated on for benign or malignant liver tumor (preoperative and 5 days postoperative) (18). Soluble TREM-1 level was significantly increased in patients suffering from HCC in comparison with those with non-malignant liver tumor/disease (ie: cyst, hemangioma, focal nodular hyperplasia) (p < 0.005) (18).

Duan et al. (10) investigated TREM-1 expression through western blot/qRT-PCR/immunofluorescence analyses in archived tissues from 322 patients who have been operated on for HCC. They demonstrated that TREM-1 was found in HCC cancer cells and tumor tissues and that high TREM-1 was significantly associated with higher recurrence and lower survival in HCC patients. Recurrence-free survival and 5-year survival rates for patients with high TREM-1 expression were 51.6% and 51.3%, respectively, in comparison with 70.9% and 62.8% for patients with low TREM-1 (p = 0.060 and p = 0.007, respectively) (10). Moreover, authors performed functional experiments which supported that TREM-1 significantly mediated invasion, proliferation, and apoptosis inhibition of HCC cells. They demonstrated that a majority of proinflammatory cytokines were significantly associated with TREM-1 level, including TNF-α, IL-1b, and MCP-1 (10).

More recently, TREM-1 expression was evaluated by IHC on 119 tissue samples from patients with HCC curative resection (19). The results they obtained are in accordance with those previously published. In their samples, the amount of TREM-1+ TAMs was significantly higher in HCC with advanced stages, which suggests that abundant TREM-1+ TAMs are engaged in malignant progression (19).

In human HCC, TREM-1 truly seems to be involved on tumor cell proliferation and invasion. Although further investigations are needed to confirm its implication in human oncology, TREM-1 could be an useful therapeutic target in human HCC.

### Inflammatory Bowel Disease-Associated Carcinoma and Colorectal Cancer

Colorectal carcinoma (CRC) is another example of cancer that is partly promoted by chronic inflammation (20). Indeed, patients with inflammatory bowel disease (IBD) are at increased risk of CRC and the long-term use of non-steroidal anti-inflammatory drugs decreases their colon cancer risk (20). In this situation, almost all the studies have been carried out on *in vivo* mouse model of DSS-induced colitis leading to the conclusion that TREM-1 inhibition reduces colitis and tumor development within animal’s colon (21, 22).

In human CRC, TREM-1 expression has been studied through a nineteen gene-based risk score (TCA19) classifier in two studies (23, 24). In a first work based on 18 matched primary human CRC samples, synchronous liver metastases and normal colonic epithelium (23), authors demonstrated that the expression level of TREM-1 assessed by RNA-sequencing was significantly higher in primary CRC tissue and their metastasis than in normal colonic epithelium (23). Those results indicate that TREM-1 expression is a predominant regulator activated during CRC tumorigenesis and may be a key event associated with CRC aggressiveness (23). Another study assessed clinical implication of TCA19 in 60 patients with stage IV CRC (24). Once more, TREM-1 was expressed at significantly higher level in primary or metastatic tumor tissues than in non-tumoral colonic tissue (assessed by RT-qPCR), suggesting that TREM-1 was related to progression and metastasis in human CRC (24).

Although those two studies have some limitations (retrospective, small number of patients), they are in accordance with a pro-tumoral role in human CRC. Further investigations are needed to confirm such observations, especially in prospective works.

Studies assessing TREM-1 and/or soluble TREM-1 (sTREM-1) expression in human digestive cancers are summarized in **Table 1**.

**Table 1:** Studies assessing TREM-1 and/or soluble TREM-1 (STREM-1)expression in human digestive cancers and the correlation with survival data.

Cancer type	Reference	Number of patients	Studied parameter	Statistical significance
Hepatocellular carcinoma	Liao et al., 2012 (17)	240	TREM-1	significantly associated with poorer OS (HR = 2.25)
	Liao et al., 2012 (17)	92	sTREM1	significantly associated with poorer OS
	Duan et al., 2014 (10)	322	TREM-1	significantly associated with increased recurrence and poorer OS (HR = 1.6)
	Wu et al., 2019 (25)	119	TREM-1	significantly associated with poorer DFS
Colorectal Carcinoma	Kim et al., 2014 (22)	18	TREM-1	significantly higher in tumoral tissues than in non-tumoral tissues
	Lee et al., 2019 (23)	60	TREM-1	significantly associated with poorer PFS

DFS, disease free survival; HR, hazard ratio; NS, not significant; OS, overall survival.

## TREM-1 in Lung Cancer

Lung cancer is the most common cause of cancer-related deaths worldwide with a 5-year survival rate for all stages of only 17% (26). TREM-1 and sTREM-1 expression were recently studied in human lung cancer, especially in human non-small cell lung cancer (NSCLC), which represents over 80% of all lung cancers (26).

TREM-1 was first investigated in lung cancer fifteen years ago, using IHC on primary NSCLC specimen from 68 surgically resected patients (27). Authors demonstrated that disease-free survival (DFS) and OS were significantly shorter in patients with high TREM-1 expression than in those with low TREM-1 level (DFS: median of 22 months versus not reached; OS: 29 months versus not reached) (27).

They also assessed sTREM-1 levels by ELISA in 4 distinct panels of pleural effusions of 65 patients (i.e., transudative pleural effusions due to congestive heart failure or postoperative reactive effusion; parapneumonic pleural effusions; tumor pleural effusions with tumoral cells and without infection; exudative pleural effusions due to neoplasia but without infection and no detectable tumoral cells) (27). They found that sTREM-1 levels were significantly higher in tumoral pleural effusions than in transudate (p = 0.017) (27).

In 2014, Yuan et al. worked on NSCLC specimen and paracarcinoma tissue from 3 patients (28). They demonstrated that TREM-1–positive cells were TAMs (28). They also performed RT-PCR and RT-qPCR on pulmonary samples from individuals with NSCLC and identified excess TREM-1 expression in human pulmonary adenocarcinoma tissues. On the contrary, TREM-1 expression was not found in normal pulmonary tissue (28).

A recent study focused on sTREM-1 levels in 164 people with lung cancer, at any stage (NSCLC: n = 137; SCLC: n = 27) (29). In patients with NSCLC, stage and sTREM-1 were prognostic values (stage: p < 0.0001, sTREM-1: p = 0.011) (29). Regarding stage IV patients (n = 75), high sTREM-1 level was a critical indicator of poorer survival (median OS: 4.8 versus 11.4 months, p = 0.009) (29). In the subgroup of SCLC patients, those observations were not confirmed (p = 0.07) (29).

Finally, an interesting work suggested there is different degrees of TREM-1 expression along NSCLC development (30). Based on analyses made of fresh tumor tissues and the matching non-tumoral tissue samples from 40 non-treated patients with NSCLC, it was shown that TREM-1 rates on tumor tissue-derived monocytes/macrophages were decreased in comparison to TREM-1 levels detected on monocytes from peripheral blood of patients suffering from NSCLC (30). Authors demonstrated that TREM-1 rates on monocytes/macrophages progressively decreased with the advancement of tumor stage and tumor invasion of lymph nodes, which suggests that weak TREM-1 expression on TAMs could be a new feature of advanced lung cancer stage (30).

TREM-1 expression in TAMs from tumoral tissues of human NSCLC was correlated with reduced DFS and OS. TREM-1 level is an independent predicator of survival in NSCLC, and might be a component of human lung cancer progression. Further investigations are needed to better understand its role in tumor immunomodulation in thoracic oncology, especially for NSCLC tumors.

Studies assessing TREM-1 and/or soluble TREM-1 (sTREM-1) expression in human lung cancer are summarized in **Table 2**.

**Table 2:** Studies assessing TREM-1 and/or soluble TREM-1 (sTREM-1) expression in human lung cancer and the correlation with survival data.

Reference	Number of patients	Studied parameter	Statistical significance
Ho et al., 2008 (26)	68	TREM-1	significantly associated with reduced DFS and poorer OS (HR = 2.72)
Ho et al., 2008 (26)	65	sTREM1	NS
Yuan et al., 2014 (27)	3	TREM-1	upregulated in NSCLC
Kuemmel et al., 2018 (28)	164	sTREM-1	significantly associated with poorer OS in NSCLC (median survival 8.5 vs. 13.3 months)

DFS, disease free survival; HR, hazard ratio; NS, not significant; NSCLC, non-small cell lung carcinoma; OS, overall survival.

## TREM-1 in Other Human Malignancies

Except for lung, liver and colon cancers, TREM-1 has been poorly studied in humans malignancies. Very recently, TREM-1 expression has been studied in other human solid tumors (**Table 3**).

**Table 3:** Studies assessing TREM-1 and/or soluble TREM-1 (sTREM-1) expression in human solid malignancies (except digestive and lung cancers).

Reference	Cancer	Number of patients	Studied parameter	Statistical significance
Xie et al., 2017 (30)	Papillary thyroid carcinoma	512 (from The Cancer Genome Atlas)	TREM-1	significantly associated with reduced PFS. significantly associated with lymph node metastasis and advanced T classification (TNM)
Kluckova et al., 2020 (31)	Glioma	63	sTREM1 TREM-1	sTREM-1 levels in the group of all glioma patients were significantly weaker than in the healthy group of patients NS
Pullikuth et al., 2021 (32)	Breast cancer	701	TREM-1	Significantly associated with inferior distant metastasis-free survival
Ford et al., 2022 (33)	Renal cell carcinoma	63 untreated patients + 531 from The Cancer Genome Atlas	TREM-1	significantly correlated with worse OS and high disease stage

PFS, progression free survival; NS, not significant; OS, overall survival.

Based on data coming from The Cancer Genome Atlas (TCGA), TREM-1 expression was compared between papillary thyroid carcinoma (n = 512) and normal thyroid tissues (n = 58) (31). TREM-1 mRNA expression was significantly higher in human papillary thyroid carcinoma tissues in comparison to non-tumoral thyroid tissues (p<0.0001) (31). The immunohistochemical results (achieved using Human Protein Atlas immunohistochemical images) demonstrated that TREM-1 protein expression was significantly upregulated in papillary thyroid carcinoma tissue compared with non-malignant tissues (31). Additionally, high TREM-1 expression was significantly associated with lymph node metastasis and with advanced T status (31).

TREM-1 and sTREM-1 expression were also recently assessed in 63 patients who underwent partial or complete resection of glioma (grade II, III and IV), in comparison to 31 healthy controls (34). In their study, authors found that sTREM-1 levels in the group of all glioma patients (especially glioblastoma subgroup) were significantly weaker than in the healthy group of patients (34). Authors explained that having a smaller amount of this decoy anti-inflammatory receptor supports the systemic inflammation. TREM-1 expression on monocytes was tightly correlated with plasma rates of proinflammatory cytokine IL-6 (34). On the opposite, they observed a negative correlation with plasma level of anti-inflammatory cytokine IL-10 (p < 0.0001), indicating that systemic inflammation has an influence on overall patient survival (34).

However, they found no significant changes when they compared TREM-1 levels on monocytes between glioma grades and between gliomas and healthy patients. In the same way, they did not observe a correlation between survival time and TREM-1/sTREM-1 expression (34). Some works support the hypothesis that neutrophils produce their own sTREM-1 in response to lipopolysaccharide challenge in a process involving *de novo* synthesis that is not accompanied by TREM-1 receptor upregulation (32).

In another study investigating the ability to detect sTREM-1 in different human solid cancers (33), authors demonstrated that sTREM-1 was detectable in the serum of 50% (7/14) patients with breast cancer (33), among 11/14 with metastasis at diagnosis. sTREM-1 expression showed a correlation to the site of metastases. Higher levels of sTREM-1 were found in the absence of pulmonary metastases (p=0.019) (33).

Recently, TREM-1 expression was also first studied in a large series of 701 human breast cancers samples (35) (obtained before neoadjuvant chemotherapy), classified in three distinct immune groups (favorable/weak or poor immune dispositions (FID/WID or PID) respectively; based on immune gene signatures). In this study, authors demonstrated that low TREM-1 levels were significantly associated with favorable response in patients with FID breast cancer (p = 0.05) (35). Moreover, TREM-1 level was determined as having the strongest statistically significant association with the lower distant metastasis-free survival (reduced response to neoadjuvant therapy). This is the first demonstration that TREM1 expression in human breast cancers has negative prognostic and therapeutic implications for breast cancer patients (35).

Soluble TREM-1 levels were also investigated in human renal cell carcinoma (RCC) (36). sTREM-1 levels were determined in a cohort of 63 untreated patients diagnosed with clear cell RCC (ccRCC). In comparison to healthy controls, sTREM-1 levels were significantly higher in patients suffering from ccRCC (mean = 265.3 pg/mL in ccRCC patients versus mean = 110.04 pg/mL in controls, p < 0.001) (36). Based on data coming from The Cancer Genome Atlas (TCGA, n = 531 ccRCC), authors also found that high TREM-1 expression was significantly correlated with worse OS and high disease stage (36).

Although TREM-1 and sTREM-1 requires further elucidation in human oncology, they might be implicated in diverse human solid malignancies and might be used as a potential biomarker for diagnosis and also disease progression. TREM-1 could also provide a new therapeutic target in different kind of human solid tumors.

## Discussion and Perspectives

Nowadays, inflammation is recognized as an essential component in cancer, playing a crucial role in cancer induction and promotion. This is largely influenced by immune cells from TME, notably TAMs. TREM-1, which is one of the most described amplifiers of MPs pro-inflammatory innate immune response, could assume a significant role in such interplay.

In this review, we summarized all the current clinical knowledges about TREM-1 in human oncology. In that situation, we may notice that TREM-1 has mainly been studied in colon, hepatocellular and lung (NSCLC) carcinoma tissues that highly expressed TREM-1 (as well as sTREM-1). Although TREM-1 has not been yet extensively studied in all kind of tumor types (ie: solid as hematological malignancies), TREM-1 should be consider as a potential biomarker in human oncology and could be used as a new therapeutic target of interest. Since the interaction between TREM-1 and DAP12 is critical for the stabilization and multimerization of TREM-1, finding TREM-1 inhibitors was a complicated process. Different TREM-1 inhibitors have been designed and studied in different murine models of malignancies associated with chronic inflammation. It was demonstrated it might confer protection against tumor progression and thus provide advantages in terms of survival through the reduction of MP inflammatory reactions. The power of this design is that it lowers, but does not fully abolish, inflammatory answers, which is crucial for tumor management. A recent Phase IIa clinical trial was conducted to assess the safety, tolerance and pharmacokinetics of the synthesized peptide nangibotide (LR12), the first pharmaceutical candidate targeting TREM-1 to be in clinical development in patients with septic shock (25). Future investigations of this agent for the treatment of cancer are pending, but this therapeutic option appears promising.

Two others TREM-1 inhibitors are also under study: LP17 peptide (a synthetic peptide blocker of TREM-1) and the GF9 peptide (a ligand-independent human TREM-1 inhibitory peptide). TREM-1 inhibition by LP17 have been studied *in vivo*, in mouse models of IBD-associated colorectal carcinoma (21). LP17 has been shown to improve the development of inflammation and tumors in the colon by providing anti-inflammatory actions. In addition, LP17 impaired intestinal epithelial growth in DSS-induced colitis (21). Blockade of TREM-1 by the delivery of the inhibitor peptide GF9 significantly abolished tumor progression in human xenograft models of non-small cell lung cancer (37). The GF9 peptide has also demonstrated therapeutic relevance in pancreatic cancer (38, 39). Its use in human pancreatic cancer xenograft mouse models caused a significant antitumoral action, which was associated with the abolition of TAM infiltrate, decreasing of serum levels of pro-inflammatory cytokines and an improvement in the animals’ survival. Moreover, GF9 therapy also significantly altered the resistance to PD-L1 inhibition, thereby enhancing its therapeutical efficiency in orthotopic HCC-bearing models (19). Those results support that specific TREM-1 inhibitors could be used as single therapy or as part of a combinatory treatment for many human solid malignancies.

Other therapeutic approaches involving TREM-1 are being studied, including the development of PY159 (40) (an afucosylated humanized anti-TREM1 monoclonal antibody) enabling the modulation of TREM-1 expression. Indeed, it was shown in *in vitro* models, that PY159 could specifically drive a selective set of pro-inflammatory cytokines and chemokines and reprogram tumor associated myeloid cells (40). PY159 tolerability and safety is under investigation in human solid malignancies (NCT04682431: an open-label, multicenter, First-In-Human, Phase 1a/1b study of PY159 in subjects with locally advanced (unresectable) and/or metastatic solid tumors that are refractory or relapsed to standard of care (including checkpoint inhibitors, if approved for that indication).

Moreover, levels on monocytes/macrophages have been shown to progressively decrease with the advancement of tumor stage and tumor invasion of lymph nodes in NSCLC, supporting that low TREM-1 expression on TAMs may be new characteristic of late stage pulmonary carcinoma. This observation provides an additional degree of complicacy to the expression of TREM-1 on other myeloid and non-myeloid cells that are implicated in anti-tumor responses

Furthermore, other *TREM* were found on human chromosome 6p21 (15), especially TREM-2 which is thought to be an important negative modulator of autoimmunity and PDC-TREM (15). Although few clinical studies of TREM2 targeting in cancer are currently being conducted, the potential for developing clinical applications of TREM2-targeted therapies remains high in the short-term future (41). Indeed, some fundamental research has identified TREM-2 as a valuable therapeutic target for cancer immunotherapy (41). Another potential role of TREM-1 concerns the regulation of apoptosis and autophagy (42), which play a double role in the regulation of senescence in normal and cancer stem cells, and of cellular responses to different therapeutical approaches (43).

More clinical studies are urgently needed to confirm TREM-1 (and TREM family) roles in the prognosis and the treatment of human cancer.

## Conclusion

Although the therapeutical application of TREM-1 inhibitors is mainly restricted to preclinical studies, modulation of the TREM-1 receptor could be a useful therapeutical application for the management of some human solid malignancies. More extensive investigations are necessary before immunotherapical approaches targeting TREM-1 may be developed in human oncology.

## Author Contributions

Manuscript writing and editing: MM, VH, AL, AT, SR, MD, and JPB. Figures design: MM. Review and approval of the manuscript: all of the authors.

## Conflict of Interest

The authors declare that the research was conducted in the absence of any commercial or financial relationships that could be construed as a potential conflict of interest.

## Publisher’s Note

All claims expressed in this article are solely those of the authors and do not necessarily represent those of their affiliated organizations, or those of the publisher, the editors and the reviewers. Any product that may be evaluated in this article, or claim that may be made by its manufacturer, is not guaranteed or endorsed by the publisher.
